# Involving patients in medicines optimisation in general practice: a development study of the “PREparing Patients for Active Involvement in medication Review” (PREPAIR) tool

**DOI:** 10.1186/s12875-022-01733-8

**Published:** 2022-05-20

**Authors:** Amanda Sandbæk, Marlene Christina Rosengaard Møller, Flemming Bro, Kirsten Høj, Line Due Christensen, Anna Mygind

**Affiliations:** 1 Department of Public Health, Aarhus University, 8000 Aarhus C, Denmark; 2 Research Unit for General Practice, 8000 Aarhus C, Denmark; 3 Department of Clinical Pharmacology, Aarhus University Hospital, 8000 Aarhus C, Denmark; 4 Department of Clinical Pharmacology, Aalborg University Hospital, 9000 Aalborg, Denmark

**Keywords:** Patient participation, Polypharmacy, Health literacy, General practice, Questionnaire, Patient-centered care, Qualitative research, Co-production, Intervention development, Denmark

## Abstract

**Background:**

Many patients have multiple health conditions and take multiple medications (polypharmacy). Active patient involvement may improve treatment outcomes and ensure patient-centred care. Yet, patient involvement remains a challenge in clinical practice. We aimed to develop and pilot test a questionnaire-based preparation and dialogue tool, the PREparing Patients for Active Involvement in medication Review (PREPAIR) tool, to encourage the involvement of patients with polypharmacy in medicines optimisation in general practice.

**Methods:**

We conducted a literature review followed by a co-production process to develop the tool: a workshop with six GPs and pilot testing, including observations and interviews, with 22 patients, three GPs and three practice staff. During this process, we made continuous adaptations to the prototype. We analysed the qualitative data thematically, focusing on the development process and mechanisms of impact.

**Findings:**

The final PREPAIR tool included five items concerning the patient’s experience of 1) adverse drug reactions, 2) excess medication, 3) unnecessary medication, 4) medication satisfaction and 5) medication-related topics to discuss with the GP (open-ended question). The applied workflow during testing was as follows; the patient completed the PREPAIR tool at home, to encourage reflection on the medication, and brought it to the GP consultation. During the consultation, the GP and the patient reviewed the patient’s responses and discussed potential medication-related problems. For some patients, the increased reflection led to worries about the medications. Still, the pilot testing showed that, when using the PREPAIR tool, the patients arrived at the clinic well prepared and empowered to speak. From the PREPAIR-supported dialogue, the GPs obtained a better understanding of patients’ perspectives and provided a more patient-centred consultation. For the patients, the PREPAIR-supported dialogue ultimately promoted an increased sense of security, satisfaction and insight into their medication, despite initial worries for some patients.

**Conclusions:**

We developed a brief tool to support active patient involvement in medication review in general practice. The PREPAIR-tool was well received by both patients and GPs and fitted well into the existing clinical practice. Our findings suggest that the PREPAIR-tool can support patient involvement during consultations and facilitate patient-centred care.

**Supplementary Information:**

The online version contains supplementary material available at 10.1186/s12875-022-01733-8.

## Background

Increasingly more patients use several medications (polypharmacy) to manage multiple health conditions [1, 2]. Polypharmacy may have significant clinical benefits [3], but every added medication increases the risk of potentially inappropriate medications (PIMs) and unintentional adverse effects [4–6]. Thus, polypharmacy may reduce the quality of life, increase the risk of hospitalisation and impose additional healthcare costs [4–6]. Estimates from Ireland have shown that half of the population above the age of 65 years receive PIMs [7].

GPs prescribe the majority of medications in many healthcare systems [8] and are often responsible for coordinating the combined medical treatment across diseases [9]. Thus, they have a pivotal role in ensuring optimal medical treatment. This role is becoming increasingly important in specialised healthcare systems that tend to focus on single diseases [9].

A medication review [10] is a commonly used method among GPs to assess medication appropriateness. During such review, it is important to consider changes in health conditions and personal preferences to optimise the medical treatment. However, optimising the patient’s medication is a complex process, and several factors related to both the GP and the patient may challenge the medicines optimisation process [11–14]. For instance, the GPs may feel obliged to adhere to medical protocols for the individual diseases and expect patient resistance to reducing or stopping some of their medications [11, 14]. Even though most patients report being willing to stop one or more medications if endorsed by the GP, many patients are unaware of the possibility to be involved in the decision-making or hesitate to share their desires for medicines optimisation with the GP [15–17]. The reasons for hesitation include expectations of insufficient support from the GP, and inadequate time and opportunity to bring up such requests during the consultation [15].

Previous studies have suggested that active patient involvement is essential for optimising medicine use and overcoming existing barriers among GPs and patients [2, 18, 19]. One of the suggested mechanisms is that involvement fosters well-informed patients who often make better choices than less-informed patients, which can ultimately lead to rational medication [2, 18, 19]. Likewise, the chronic care model, which is a widely accepted framework for providing care for people with chronic conditions in a primary care setting, states that optimal chronic care is achieved when a prepared, proactive practice team interacts with an informed, activated patient [20]. Yet, patient involvement in the consultation is generally sparse [21], especially among patients with low health literacy [22]. Actions to increase patient involvement by improving their knowledge of their medication and efforts to put such knowledge into practice may enable patients to exert greater control over their medical treatment [23]. Accordingly, developing tools that provide the GP with feedback from patients on their goals and preferences have been suggested as a possible strategy for medicines optimisation [24, 25].

To accommodate this need for tools, a rapid-cycle participatory design could be used, as it has proven suitable for intervention development [26, 27]. This design involves stakeholders and end-users in the process through small development cycles and continuous exchange between practice and research. Such design is known to produce interventions that are relevant to patients and implementable in daily clinical practice [27–30].

### Aim

With inspiration from the concept of health literacy, we aimed to develop and pilot test a questionnaire-based preparation and dialogue tool, the PREparation of Patients for Active Involvement in medication Review (PREPAIR) tool, to encourage the involvement of patients with polypharmacy in medicines optimisation in general practice.

## Methods

### Setting

Danish healthcare is mainly funded by public taxes, and all residents have free-of-charge access to services. Annual chronic care consultations are provided by the general practice to patients with one or more chronic conditions but usually focus on one specific diagnosis. The organisation of these consultations varies across clinics depending on e.g. clinic characteristics such as size and ownership [31]. However, they usually involve two patient encounters: a consultation with practice staff (e.g. blood sampling and medication reconciliation) and a consultation with the GP (e.g. test results, holistic medication review and motivational conversation) [32, 33]. The PREPAIR tool was developed to fit the workflow of the annual chronic care consultation.

### Theoretical framework

The study was based on the concept of health literacy, which framed the data analysis, including the researchers’ perceptions of the mechanisms of impact. Health literacy can be defined as a person’s competencies and resources to access, understand, appraise and use health information [34]. Health literacy is linked to health outcomes through, among other factors, patient-provider interaction [35]. Such interactions are influenced by the individual patient’s knowledge, beliefs and participation in decision-making as well as the provider’s communication skills, teaching ability, time and ability to facilitate patient-centred care [35]. Thus, our theoretical preconception was that introducing a preparation tool was likely to improve the patients’ health literacy and facilitate patient-provider interaction, which is known to enhance patient engagement and ultimately improve health outcomes [22, 35].

### Study design

**Fig. 1:**
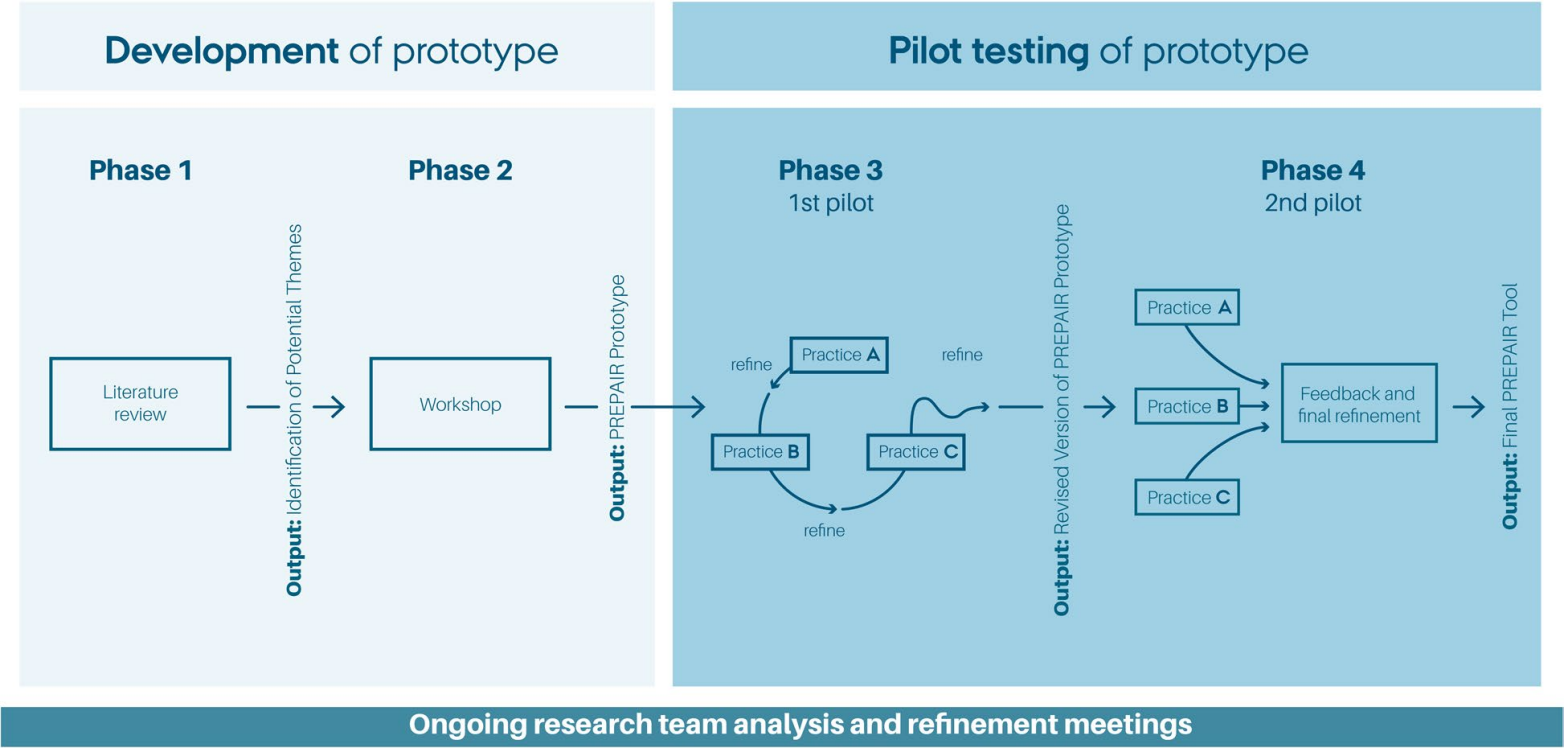
Development phase of the PREPAIR tool

The study took place from April 2019 to June 2020. We used a co-producing participatory approach and small rapid cycles of development, adaptation and evaluations with a continuous exchange between researchers and stakeholders (Fig. 1). GPs, staff and patients participated in the development process, thereby ensuring that the intervention would fit the general practice setting [36].

The PREPAIR tool was developed in four phases: literature review, workshop, first pilot testing and second pilot testing. Insights from earlier phases were incorporated to make adaptations. These iterative processes of constant exchange between practice and research allowed for continuous evaluations and adjustment.

### Participants

For the workshop, we recruited six GPs from the research team’s network and two networks in the Central Denmark Region for general practice clinics with a special interest in and dedication to quality development. The prototype was tested in three GP clinics recruited from these two networks, and one of these GPs also participated in the workshop. One GP and one practice staff member from each of the three pilot clinics participated in both rounds of pilot tests. The participating GPs and practice staff represented geographic and demographic variation in practice type, age and gender. Different patients were included in the pilot testing: 12 patients were included in phase three and 10 in phase four. Inclusion criteria for patients in the pilot testing were: age ≥ 18 years, current use of a minimum of five regular medications and a scheduled annual chronic care consultation. Of the interviewed patients, five were women and 13 were men, and they were between the age of 57 and 85. For the remaining four patients, we did not obtain information about sex and age, since they were not included in interviews and/or observations. To avoid compromising the recruitment, we did not ask the clinics to collect additional information on the participating patients. An overview of the informants’ participation in the development phases is presented in Table 1.

**Table 1:** Overview of informants’ participation in the development phases

Clinic no	Practice type	Informants		
		**Workshop**	**Pilot 1**	**Pilot 2**
1	Singlehanded	GP1	GP1, S1, P1, P2	GP1, S1, P13, P14, P15, P16
2	Group	GP2		
3	Group	GP3		
4	Group	GP4		
5	Singlehanded	GP5		
6	Group	GP 6	GP7, S2, P3, P4, P5, P6, P7 ^1^	GP7, S2, P17
7	Group		GP8, S3, P8, P9, P10, P11^1^, P12^1^	GP8, S3, P18, P19, P20, P21, P22 ^1^

*GP* General practitioner, *S* Staff, *P* Patient, ^1^No interview

### Data collection and analysis

#### Phase 1: Literature review

To identify existing questionnaires or tools for unveiling patient attitudes or experiences with medicines, we performed a literature search. The literature review was conducted in April 2019 in the PubMed database as a block search combining search terms for patients, intervention, context, outcome and item [see Additional file 1]. Based on the identified literature, the researchers performed a rough compilation of similar questions and themes, thereby producing a gross list of potential items to include in the prototype of the PREPAIR tool. Then, the researchers condensed the list based on considerations about relevance, acceptability and potential to foster meaningful dialogue.

#### Phase 2: Workshop

The literature search was followed by a workshop (held in November 2019) with GPs who delivered input for the prototype based on their experiences and perspectives. During the workshop, we presented the list of potential themes to the GPs, and they were guided through the co-production process, including discussions of potential themes, suitability in daily clinical practice and considerations of the context of the chronic care consultation. After the workshop, the discussions were analysed through the use of a rapid analysis approach [29]. The workshop was recorded on video, which allowed the researchers to review the recordings to resolve dilemmas and achieve consensus on the prototype.

#### Phase 3: First pilot testing

As part of the first pilot testing, the clinics were introduced to the prototype by one of the researchers. This allowed the GPs and the practice staff to pose questions and challenge the prototype content and workflow before testing with their patients. In this phase, GPs, staff and patients in the three clinics (Table 1) were asked to consider potential improvements while using the prototype. The clinics were enrolled in a stepped process, whereby improvements obtained from the pilot test in one clinic were incorporated into the prototype tested in the following clinic (see Phase 3 in Fig. 1). The process was closely monitored by the researchers and involved continuous adaptations. To gain insight into the feasibility and acceptability of the intervention content and workflow, we conducted focused observations of patient encounters with practice staff and GPs. Furthermore, informal interviews with patients, GPs and practice staff were conducted before or after the encounters. A few patients declined to participate in the follow-up interviews. Field notes were taken continuously. After each pilot testing, the prototype was revised based on rapid analysis. An overview of the data collection is provided in the additional files [see Additional file 2]. Phase 3 was undertaken from November 2019 to April 2020.

#### Phase 4: Second pilot testing

During the second pilot testing, the participants were asked to consider potential improvements and to pay special attention to the mechanisms of impact. In this phase, the pilot tests were conducted concurrently in the three clinics, and adaptations were only made at the end of the pilot testing (see Phase 4 in Fig.1). Because of the COVID-19 lockdown (spring 2020), no participant observations were made during the second pilot testing. Instead, updates were obtained from GPs and practice staff, and feedback interviews were conducted at the end of each pilot testing via e-mail, telephone or video communication. Patient perspectives and inputs were explored through short telephone interviews, which were conducted shortly after their GP consultation. One patient did not participate in a follow-up interview. Notes were taken during and immediately after the telephone interviews. Feedback interviews with the GPs and practice staff were recorded. Subsequently, the prototype underwent the final adaptation. The specifications of the data collection are illustrated in the additional files [see Additional file 2]. Phase 4 was undertaken from May 2020 to June 2020.

### Thematic analysis

After the final adaptation of the prototype, we conducted a thematic analysis of all data, i.e. workshop recordings, feedback interviews, observation notes, field notes, interview notes and e-mail correspondences. We used open, axial and selective coding. First, we analysed all data, either line by line or in small sections, to identify meaning units. Subsequently, we refined the categories and themes in an iterative process of reading and systematically reviewing the data and initial codes until patterns emerged. The theoretical framework guided the analysis process for the mechanisms of impact [34, 35]. Data were clustered in categories under two overarching themes: 1) development and adaptations of the tool (e.g. selection and deselection of questions and response options, completed adaptations and reasons for these, barriers and facilitators for implementation) and 2) mechanisms of impact (e.g. patient reflections, GP-patient dialogue, patient- and GP-reported outcomes).

## Results

### Developing and adapting the tool

In the literature search, we were able to identify 30 relevant tools (communication aid or questionnaires) of which 25 were available in full [37–61]. From these tools, we identified a gross list of 386 items, which was reduced to a list of questions exploring 22 themes. These were reduced to five themes based on workshop discussions. During these discussions, the GPs considered each question for relevance and appropriateness, taking into account their everyday clinical work and the purpose of the chronic care consultation. For instance, regarding the deselection of the theme ‘medication adherence’, one GP said:

*“I’m hesitating because people can reply anything to satisfy the doctor. We may risk catching people with their pants down as we may see something else in their blood tests”* (GP 1)

In the discussions, the GPs emphasised the importance of a short format to avoid lengthy time consumption in the consultation and requested a readily understood tool.

During the pilot testing, the prototype was continuously revised, and several edits were made to improve the wording, response categories, item order and layout. The five themes identified in the development phase were maintained, and no additional themes were added.

The results of each step leading to the final tool are shown in an additional file [see Additional file 3].

The final PREPAIR tool used a three-point Likert scale and included five items (adverse drug reactions, excess medication, unnecessary medication, medication satisfaction, and an open-ended item on medication-related topics for discussion) (see Table 2). A layout version of the PREPAIR tool is available in the additional files [see Additional file 4].

**Table 2:** Items and response options in the final PREPAIR tool

Item no	Statements	Response options
1	I experience adverse drug reactions of the medication that bother me significantly	Agree/Neutral/Disagree
2	I sometimes think that I get too much medication	Agree/Neutral/Disagree
3	I think that I might get some medication that I do not need	Agree/Neutral/Disagree
4	I am overall satisfied with my current medication	Agree/Neutral/Disagree
5	Is there something about your medication that you would like to discuss with the GP? If yes, please elaborate:	Yes/No Open-ended

The PREPAIR tool was intended as an instrument allowing patients with polypharmacy to engage in discussions about their medication. However, the pilot testing revealed that some patients found it difficult to complete the PREPAIR tool due to memory loss, poor literacy or low language proficiency. Moreover, as annual chronic care consultations comprise discussions of other aspects than medication, one GP requested a broader focus of the PREPAIR tool to reach beyond medication and include more aspects of patient health and healthcare use. 

**Fig. 2:**
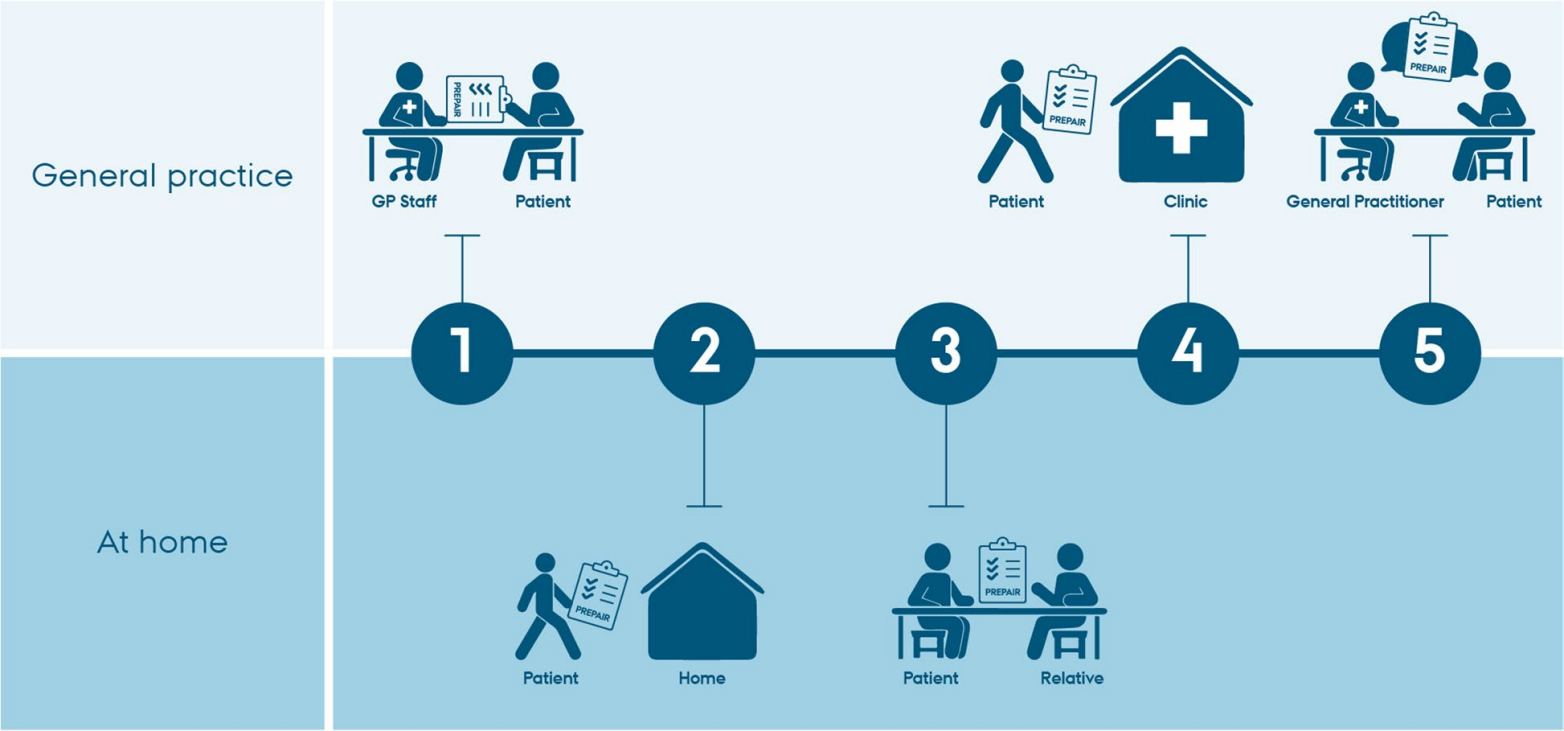
Workflow for the PREPAIR tool in chronic care consultations in general practice

During the development and pilot testing of the prototype, the PREPAIR tool was found suitable to include in the annual chronic care consultation. Thus, the practice staff introduced the prototype to the patients at their first encounter and encouraged them to complete the tool at home (sometimes together with a relative) and bring it for the GP consultation. A few patients forgot to bring the printed PREPAIR tool to the clinic. In these cases, the clinics handed out a blank tool for the patient to complete before the GP consultation. The workflow is illustrated in Fig. 2.

## Mechanisms of impact

**Fig. 3:**
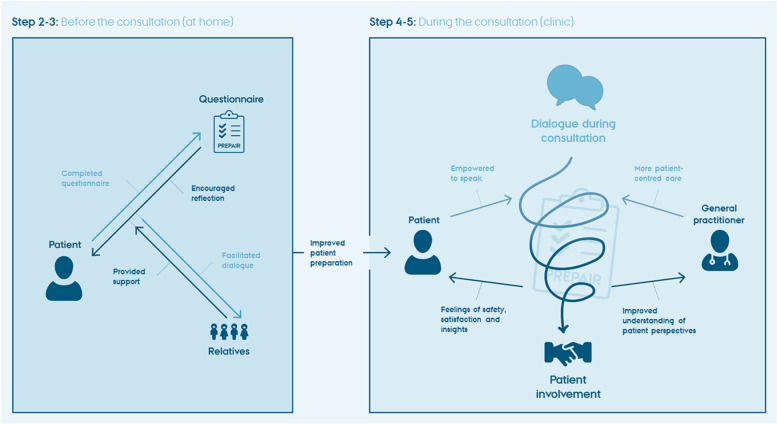
Mechanisms of impact of the PREPAIR tool

From our analysis, we identified several impact mechanisms on how completing the PREPAIR tool affected the patient preparation and the patient-provider interaction (see Fig. 3). These mechanisms are identified based on the theoretical framework and elicited in the following sections under two main categories: before and after the consultation.

### Before the consultation

#### Preparation facilitated reflections and awareness

When evaluating the PREPAIR tool, some patients mentioned that using the tool generated reflections about their own experiences, beliefs and needs, which raised their awareness of own perspectives and opinions. A patient explained:

*”It [using the tool] makes you think a little about the pills you get – is it really necessary? Is there something that you aren’t satisfied with? […] I think a lot [of people] could benefit from that.”* (P 21)

Some patients completed the PREPAIR tool on their own, while others did it together with a close relative, e.g. their spouse. In some cases, completing the tool fostered an important dialogue with the relative, which facilitated further reflections and higher awareness about medications.

For some patients, reflecting on their medication was a novel experience. In these cases, filling in the PREPAIR tool triggered a new awareness of potential uncertainties about their medication. A patient elaborated:

*”I have sleep problems, and perhaps it is due to adverse drug reactions, I come to think about. I would not have asked the doctor if I had not filled out the [PREPAIR] questionnaire. […] You become aware of something that you could ask the doctor about*.” (P 14)

Thereby, the reflections generated from the question about adverse drug reactions combined with the open-ended question on topics to discuss with the GP enhanced the patients’ articulation of these uncertainties. The process of completing the questionnaire supported the patients’ feeling of being prepared to engage in dialogue with the GP about their medication. Some GPs noticed that the patients seemed better prepared and appreciated that the patients actively reflected on their medication before the consultation.

#### Unnecessary worries

Although reflections were primarily described as beneficial, some patients pointed out that these new reflections might foster concerns. For some people, being confronted with the PREPAIR tool induced uncertainty and doubts about the current medical treatment. One patient said:

*”I think that I got a lot of pills for the same things, blood anticoagulants and arrhythmia [medications]. Can it really be true that I need to take all this? I had not thought about it until I got the [PREPAIR] questionnaire. […] The question is a little strange because I expect it to be under control.”* (P 13)

For others, the mere introduction of new reflections was unwanted, as it created a renewed awareness of the disease in their everyday lives and nurtured underlying disease anxiety. A patient said:

*”I don’t want [to engage in] all that talk about disease. It must not take a strong presence in my life. I am so f…. nervous before such a visit to the doctor.”* (P 2)

Similarly, a GP raised concerns that enhanced patient preparation might evoke expectations that the GP would be unable to fulfil and thus cause more harm than good. The GP explained:

”*It’s a dialogue I want to have with the patient. If no [medication] changes should be made, then I don’t want the patient to think about possible changes before [the consultation].*” (GP 6)

This quote illustrates a standpoint that, from the GP’s point of view, a clinical indication is needed to initiate medication changes while the antecedent for using the PREPAIR tool is that patients’ perspectives alone can motivate medication changes.

### During the consultation

#### Patient empowerment

Without the PREPAIR tool, some patients explained that they would take a more passive role in the annual chronic care consultations. Many patients found that the purpose of this consultation was to receive test results. Therefore, the consultation was not suitable for addressing worries that they would like to discuss with the GP. A patient said:

*”I don’t usually bring up my worries [at the consultation].”* (P 20).

However, bringing the PREPAIR tool to the consultation provided the patients with a physical manifestation of their perspectives, which served as a communication device to articulate their needs, beliefs and emotions. Thus, the PREPAIR tool provided the patients with an opportunity to speak and empowered them to do so.

During the consultations, we observed that some patients seized the opportunity to present their opinions and elaborate on specific themes. One patient stated:

*”I don’t want to get too much medicine, then I’d rather have a little pain. We also talked about that at the consultation.”* (P 17)

Hence, the PREPAIR tool encouraged the patients to express their preferences and priorities.

#### Patient-centred care

Some GPs mentioned that there was a limited focus on patient perspectives in the discussion of medication in the usual annual chronic care consultation. They often found that conveying test results should be prioritised during these consultations. A GP expressed it this way:

*“You need to pull yourself together to ask [about the patient’s perspective] because you are pressed for time – you must give the results of the tests, that is the first priority.”* (GP 3)

In general, the GPs perceived patient involvement as a challenge in daily routine care. Some GPs voiced that they often had a predefined treatment plan before the consultation, which challenged the involvement of patients. A GP exemplified in the context of annual care consultations for type 2 diabetes:

*”I need to practice asking [about the patient’s perspective] because, you see, I know that diabetes involves all these medications, so I just keep going, right?”* (GP 2)

However, when introducing the PREPAIR tool in the consultations, we observed that the GPs turned their attention away from the computer screen by physically turning their chair to face the patients when asking the patients about the PREPAIR tool responses. This was also recognised by some of the patients. One reflected on the consultation:

”*It was a good dialogue with the doctor. The previous [doctor] just sat there, staring at the screen.*” (P 21)

Thus, using the PREPAIR tool nudged the GPs to give room for the patients’ perspectives on medication in addition to test results and their clinical implications. According to patients and GPs, this influenced the course of the consultation and supported a more patient-centred approach to medicines optimisation. A GP elaborated:

*“I think perhaps that it’s this thing about saying ‘oh, well okay, let’s try to use your experience as our starting point’.”* (GP 8)

Likewise, a member of the practice staff noticed that, in her experience, the GP consultations became more focused on what was important for the patients.

When patients appeared insecure, the GPs used the PREPAIR tool to encourage these patients to communicate their wishes. This was done in a consultation, where the patient thought that no changes could be made and therefore found it pointless to present his wishes. In this case, the PREPAIR tool assisted the GP in encouraging the patient to elaborate on his interest in deprescribing. The GP explained:

*“I had one [patient], and he says, ‘I think that I get too much medicine, but I realise that I cannot do without it’. But then I say, ’which medicine would you like to get rid of, if possible?’ [Then the patient says,] ’Well, it would probably be this one’.”* (GP 8)

#### Improved GP understanding

The GPs repeatedly emphasised that prior knowledge of the patient played an important role in evaluating the patient’s medication. However, the GPs were aware that they sometimes made unsubstantiated assumptions of patient preferences based on this relation. One GP explained:

*”Sometimes we think that having to take 12 medications is hard for the patient, but perhaps it is not.”* (GP 6)

Yet, one GP described patient concerns as sometimes surprising because the rationale for the treatment plan was obvious to the GP:

*”They [the patients] have some questions about, ’why do I get two different kinds of medications for blood pressure?’, for example. I don’t think about it at all, that there could be anything to be in doubt about. Then they might think that it could be an actual mistake, right?”* (GP 1)

Thus, in some cases, the dialogue based on the completed PREPAIR tool provided the GPs with a new understanding of the patients’ preferences and concerns. We observed that the PREPAIR tool enabled patients to convey their messages in a language that the GPs could understand.

In other cases, the dialogue did not bring any new information or raise concerns about the medication. Nevertheless, the feedback in terms of the patients’ positive experiences and general satisfaction was perceived as valuable in itself by the GPs. The observations showed that the review of the completed PREPAIR tool took a few minutes in the consultation when the patients had no problems or concerns related to their medication, which justified the use according to the GPs.

#### Feelings of safety, satisfaction and insights

The dialogue with the GP diminished patients’ preliminary worries and insecurity from completing the PREPAIR tool. Instead, the patients mentioned a sense of medication safety, improved understanding of their medication and better communication, which was perceived to outweigh the preliminary worries. Some patients believed that the GP-patient dialogue based on the completed PREPAIR tool may prevent medication errors and provide renewed reassurance of medications. A patient said:

*“I got an explanation of why I need several mediations for the same thing. So, now I feel reassured about getting it.”* (P 13)

Moreover, one patient explained that indicating a willingness to discuss the amount of medication had made the GP more thorough in the communication, which the patient appreciated. Other patients mentioned that they had obtained better insight into their medical treatment due to improved communication with the GP. A patient said:

*”I was explained about how my heart has been affected by the thrombus. The way of talking about it was better [than usually].”* (P 2)

Increased involvement was described as valuable for patients who found it difficult to articulate their perspectives without the PREPAIR tool. Likewise, the GPs found that using the PREPAIR tool had improved patients' understanding of their medications. A GP said:

*“It [the dialogue based on the completed PREPAIR tool] has perhaps given the patient a better understanding of why they get the medicine, and I actually think that’s really fine.”* (GP 1)

## Discussion

In this study, we developed a new questionnaire-based tool to encourage patient preparation and GP-patient dialogue about medications in connection with a medication review. The tool fitted well with the existing workflow in general practice and was perceived as feasible and meaningful to both GPs, clinic staff and patients. When preparing for the consultation using the PREPAIR tool, the patients reflected on their medical treatment and became more aware of their perspectives. For some patients, this also led to worries about the medications. During the consultation, the PREPAIR tool functioned as a shared communication nexus through which the patient was empowered to speak and the GP took a more patient-centred approach than usual. Ultimately, the GPs gained a better understanding of the patients’ perspectives, and the patients experienced an increased sense of safety, satisfaction and insight into their medical treatment, despite initial worries for some patients.

From the existing literature, we found that questionnaires exist for illuminating medication-related experiences, preferences and attitudes [38, 40–62]. However, they are primarily screening or measuring tools developed for research purposes [40–60, 62]. A few clinical tools or interventions have been developed for engaging patients in decision-making about medications in general practice [37–39, 63–69]. A barrier to the routine use of these tools or interventions is that they are designed to require additional or lengthy consultations [37, 38, 67] or have a broader treatment scope [39, 67–69]. In contrast, the PREPAIR tool is a brief clinical tool; it was developed to fit the existing general practice setting and workflow, and it was specifically designed to improve the dialogue on medications. Some of the available tools are intended for preparing the GP for the dialogue [37, 38, 65–67]), whereas the PREPAIR tool focuses on the patient’s preparation and on eliciting the patient’s agenda. Further, the PREPAIR tool differs from the existing tools by constituting a physical artefact in the consultation, which can act as a boundary object between the GP and the patient and create a nexus for communication [70]. Finally, some tools merely involve an educational element with no scheduled follow-up [63–66, 68, 69]. In contrast, the workflow of the PREPAIR tool includes a follow-up consultation with the GP, in which medication-related issues and potential concerns can be resolved.

We found that introducing the PREPAIR tool supported the patients to take a more active role in the consultation, which improved the communication about medication according to both GPs and patients. Usually, GPs take the initiative for the annual chronic care consultation; the consultation is often steered by a predominately GP-set agenda, ultimately making the patient engagement sparse. Accordingly, existing research suggests that a significant part of the patients’ communication consists of minimal acknowledgement tokens and that patients often leave the consultation with unvoiced agendas [21, 71]. Moreover, in our study, the GPs expressed that without the PREPAIR tool they sometimes struggled to involve patients in their medication, especially when the clinical indications for medication changes were sparse. This also corresponds with previous research, which indicates that GPs need support to involve patients more systematically in their medical treatment [72, 73]. Our results suggest that routine use of the PREPAIR tool could be a way to facilitate systematic patient involvement and that it may provide a valuable instrument to support patients in voicing their perspectives, thereby bridging the gap between GP and patient agendas.

Some patients in our study preferred not to reflect on their medications. The diversity in patient preferences for involvement is important to keep in mind. Moreover, the PREPAIR tool might not be suitable for all patients. Our findings showed that some patients with linguistic or cognitive impairments found it difficult to complete the PREPAIR tool by themselves. A workflow requiring patients to complete the PREPAIR tool at home could therefore exclude some patients from using the tool. Yet, involving practice staff or relatives in completing the PREPAIR tool may enable these patients to use the tool. Such an approach may also benefit the patients by enabling them to discuss their perspectives in a safe environment before the consultation, thereby empowering them to present their perspectives in the consultation with the GP.

### Future research

The role of relatives in completing the PREPAIR tool was not explored in this study, as well as when and how to appropriately involve them in the medicines optimisation process. However, these aspects represent important areas that warrant further investigation.

Moreover, an important focus for future research would be to explore the impact of using the PREPAIR tool on patients with low social status. These patients more often find it difficult to communicate personal values and preferences to healthcare professionals, e.g. due to low health literacy and limited communicative skills [22, 74], and our findings indicated that the PREPAIR tool might contribute to improved health literacy. This indicates that the PREPAIR tool could be particularly beneficial for these patients. Correspondingly, rethinking the accessibility and the user-friendliness of the PREPAIR tool might enhance the ability of vulnerable patients to engage in the medicines optimisation process, which could contribute to more equality in healthcare. This may provide a broader scope for the PREPAIR tool, e.g. in other settings involved in medicines optimisation such as in care homes or at hospital discharge.

### Strengths and limitations

An important strength of this study is the rapid-cycle participatory approach that allowed us to develop a pragmatic (only five items) and feasible tool that is tailored for the end-users and existing workflows in general practice. The strong focus on the relevance and feasibility of the PREPAIR tool may reduce barriers to usage, increase its acceptability and ultimately improve the implementation. Using rapid analysis implied continuous revisions of the tool. To ensure the validity of the results, we continuously documented and thoroughly discussed our choices within the cross-disciplinary research team. The study also has limitations. In the second development phase, only GPs participated. Inviting patients or staff members for this process could have resulted in the selection and deselection of different questions. Moreover, the participating test clinics were deeply involved in the development and represented a selected group of GPs with a particular interest in quality development, which may have induced a sense of ownership. Exploring the use of the PREPAIR tool in different clinics without former involvement could add new perspectives. Also, involving more than three clinics and more patients e.g. in a feasibility study would strengthen the reliability of the findings. Finally, it is important to keep in mind that the PREPAIR tool is limited to a medication focus which only constitutes one aspect of the chronic care consultation. Ideally, the patients should be involved in all phases of the consultations.

## Conclusions

In this study, we developed the PREPAIR tool; a new questionnaire-based 5-item preparation and dialogue tool to encourage active patient involvement in the GP-patient dialogue about medications during a medication review. We found that the PREPAIR tool fostered more pre-consultation patient preparation, patient empowerment and patient-centeredness by GPs. Using a PREPAIR tool as a physical artefact provided a shared communicative nexus. In combination, these mechanisms contributed to enhanced patient involvement during the consultation, which improved GP understanding of patient perspectives and gave patients a feeling of safety, satisfaction and insights into their medication. Despite initial worries in some patients, the PREPAIR tool was well received by both patients and GPs. The tool appears to fit well into the existing format of annual chronic care consultations in general practice, and using the tool requires only limited time during consultations. Thus, the PREPAIR tool may provide a feasible instrument to support patient involvement and facilitate patient-centred care in medicines optimisation.

## Fundings

This work was supported by TrygFonden and the Committee for Quality Improvement and Continuing Medical Education (KEU) of general practice in the Central Denmark Region. AS was granted a PhD scholarship from the Danish Research Foundation for General Practice and the Graduate School of Health at Aarhus University. The funding sources had no role in the design, analysis and interpretation of the data, or writing of the study.

## Supplementary Information

**Additional file 1:** Overview of literature search

**Additional file 2:** Overview of data collection in phase 2-4

**Additional file 3:** Details form the development and pilot testing of prototype

**Additional file 4:** The final PREPAIR tool

## Data Availability

The datasets generated and analysed during the current study are not publicly available due to confidentiality reasons or ethical restrictions but are available from the corresponding author on reasonable request.

## Abbreviations

GDPR: General Data Protection Regulation
GP: General practitioner
MOSAIC: Medicines Optimisation – a Systematic Approach In primary Care
P: Patient
PIM: Potentially Inappropriate Medication
PREPAIR: PREparing Patients for Active Involvement in medication Review
S: Staff
