# Supplementary material for: Involving patients in medicines optimisation in general practice: a development study of the “PREparing Patients for Active Involvement in medication Review” (PREPAIR) tool
Source: BMC Prim Care. 2022 May 20;23:122. doi: 10.1186/s12875-022-01733-8 (PMC9121082; doi:10.1186/s12875-022-01733-8)
Supplement: Supplementary file 1 — Additional file 1: Overview of literature search [file 12875_2022_1733_MOESM1_ESM.pdf]

**ADDITIONAL FILE 1:** OVERVIEW OF LITERATURE SEARCH

Search terms used for the literature review in PubMed database

| | | | AND | | |
|---|---|---|---|---|---|
| | Facet: Patient | Facet: Intervention | Facet: Context | Facet: Outcome | Facet: Items |
| OR | Chronic patients | Patient involvement | Primary care [MeSH] | Deprescrib* | Tool |
| | Chronic patient | Patients' attitudes | Primary care | Medication reconciliation [MeSH] | Questionnaire |
| | Multimorbidity | Patient reported measure* | General practice | Medication reconciliation | Instrument |
| | Polypharmacy [MeSH] | Patient reported experience* | Family medicine | Medicine review | Survey |
| | Polypharmacy | Patient reported outcome* | Family doctor | Medical review | Form |
| | Multiple chronic conditions | Patient perspective | Family doctors | Medication review | Decision aid |
| | Multiple chronic conditions [MeSH] | Patients perspective | Primary health care | Medication optimization | Assistance |
| | Potentially inappropriate medicine | Patient reported outcome measures [MeSH] | Phc | Potentially inappropriate medication list [MeSH] | Patient health questionnaire [MeSH] |
| | Chronic disease | Patient reported outcome measures | Family physician | Potentially inappropriate medication | Patient health questionnaire |
| | Chronic diseases | Patient outcome assessment [MeSH] | Family physicians | | Surveys and questionnaires [MeSH] |
| | Chronic illness | Patient outcome assessment | General practitioner | | Surveys and questionnaires |
| | Chronically ill | Patient experiences | GP | | |
| | | Patient participation [MeSH] | General physician | | |
| | | Patient participation | General physicians | | |
| | | | Physicians, primary care [MeSH] | | |
| | | | PHC | | |
