# Supplementary material for: Involving patients in medicines optimisation in general practice: a development study of the “PREparing Patients for Active Involvement in medication Review” (PREPAIR) tool
Source: BMC Prim Care. 2022 May 20;23:122. doi: 10.1186/s12875-022-01733-8 (PMC9121082; doi:10.1186/s12875-022-01733-8)
Supplement: Supplementary file 2 — Additional file 2: Overview of data collection in phase 2-4 [file 12875_2022_1733_MOESM2_ESM.pdf]

ADDITIONAL FILE 2: OVERVIEW OF DATA COLLECTION IN PHASES 2-4

Data collection		
**Phase**	**Data type**	**Participants**
Phase 2<br>*Workshop*	Video recording	GP1, GP2, GP3, GP4, GP5, GP6
Phase 3<br>*1st pilot*	Observations	Clinic 1: 3 observations of consultations (GP1+P1, GP1+P2, S1+P2)<br>Clinic 2: 5 observations of consultations (GP7+P3, GP7+P4, GP7+P5, GP7+P7, S2+P7)<br>Clinic 3: 2 observations of consultations (GP8+P8, S3+P8)
	Field notes and informal talks	Clinic 1: GP1, S1, P1, P2<br>Clinic 2: GP7, S2, P3, P4, P5, P6. P7<br>Clinic 3: GP8, S3, P8
Phase 4<br>*2nd pilot*	Interviews (zoom or phone)	Clinic 1: P13, P14, P15, P16, GP1,S1<br>Clinic 2: P17, GP7, S2<br>Clinic 3: P18, P19, P20, P21, GP8
	E-mail feedback	Clinic 1: GP1

GP: General Practitioner, S: Staff, P: Patient
