# Supplementary material for: Involving patients in medicines optimisation in general practice: a development study of the “PREparing Patients for Active Involvement in medication Review” (PREPAIR) tool
Source: BMC Prim Care. 2022 May 20;23:122. doi: 10.1186/s12875-022-01733-8 (PMC9121082; doi:10.1186/s12875-022-01733-8)
Supplement: Supplementary file 3 — Additional file 3: Details form the development and pilot testing of prototype [file 12875_2022_1733_MOESM3_ESM.pdf]

ADDITIONAL FILE 3: DETAILS FROM THE DEVELOPMENT AND PILOT TESTING OF PROTOTYPE

Developing the prototype

Phase 1: Literature review

The search blocks were combined stepwise, and no filters were applied. We included all articles published in English or Scandinavian languages. Included articles concerned the development or validation of instruments for assessing aspects relating to any experiences or perspectives on medicines. The PubMed literature search gave 469 hits, which were screened by title and abstract. A total of 39 articles were read in full, and of these, eighteen articles met the inclusion criteria as they concerned specific tools or questionnaires on patients' beliefs, attitudes, perspectives or similar concerning medicines. In total, we were able to identify 30 relevant tools (communication aids or questionnaires) of which 25 were available in full [1-25]. This resulted in a gross list of 386 items. These items were thematised, and duplicates were deleted. The items covered 37 themes to consider for inclusion in the following workshop.

In the first condensation made by AS and AM, the list of potential questions were reduced to 79 items, still covering 36 themes. This list was then considered by the entire research group, who further condensed the list of questions to 22 items covering the following six themes: personal information, experiences with medicine, concerns about medicine, difficulties related to medicine, needs of aid related to medicine and wishes for treatment (details presented in table 1).

Phase 2: Workshop with GPs

During the workshop, the participants discussed the list of potential themes and items and rated them by their ability to enable prosperous dialogue with patients. The number of questions was reduced as the GPs preferred a concise and brief questionnaire. Based on their discussions and perspectives, the research group adjusted the questionnaire and reduced the number of items to five, which were culturally translated into Danish, and the exact wording was selected. Based on the existing literature, the research group decided on the response options and scales before initiating the first pilot test.

Table 1: Changes made at phase 1-2 during the development of the prototype

Phase	Phase 1		Phase 2
	Step 1: Literature search	Step 2: Condensation	Workshop with GPs
**Revisions during phase**		Coding by the researchers and condensation into specific themes. Sort out of least relevant themes.	Omitting least relevant themes and wordings. Development of first draft questionnaire.

**Format after phase**	List of 386 questions (covering 37 themes)	List of 22 themes	List of five questions<br>Reply on a five-point Likert scale (highly agree, agree, neither agree nor disagree, disagree, highly disagree)
**Content after phase**	• Information about the patient<br>• General satisfaction with treatment<br>• Open questions/ coaching questions<br>• General opinions of medication<br>• Medication dependence<br>• Concerns<br>• Misunderstandings<br>• Health literacy<br>• Knowledge about medication<br>• Wishes/hopes<br>• Self-efficacy<br>• Adherence/compliance<br>• Medication effects<br>• Factors associated with adherence/compliance<br>• Numbers/ quantity of drugs<br>• Adverse drug reactions<br>• Symptoms<br>• Use of alternative/over-the-counter medication<br>• Medication-related burden: in general<br>• Medication-related burden: format/management of drugs<br>• Medication-related burden: planning and organisation<br>• Medication-related burden: everyday life/ activities<br>• Medication-related burden: economic considerations<br>• Medication-related burden:: social considerations<br>• Treatment burden<br>• Attitudes towards medication among families and friends<br>• Support (emotional and practical)<br>• Expectations of GP's opinions<br>• Supportive tools<br>• GP-patient relation/ communication<br>• Satisfaction with information about medication<br>• Satisfaction with information about medication problems<br>• Experiences with medication changes<br>• Opinion on importance of medication changes<br>• Expectations for medication changes<br>• Drug monitoring<br>• Follow up	• Living conditions<br>• Educational background<br>• Adherence<br>• Over-the-counter medicines<br>• Other treatments<br>• Satisfaction with current treatment<br>• Knowledge about medicines<br>• Adverse drug reactions<br>• Concerns about the effect of the medication<br>• Concerns about adverse drug reactions<br>• Discontinuation of medications<br>• Suspicion of unnecessary medication<br>• Suspicion of wrong medication<br>• Difficulties with taking medication<br>• Routines or daily activities<br>• Financial constraints<br>• Acquisition of medicines<br>• Medication management assistance<br>• Support or assistive devices<br>• More involvement<br>• More dialogue/ knowledge<br>• Discontinuation of medicines	How much do you agree with the following statements?<br>• I am overall satisfied with my current medication<br>• I sometimes think that I get too much medication.<br>• I experience adverse drug reactions of the medication that bother me significantly.<br>• I have a sense that I might get some medication that I do not need.<br>• Is there something about your medication that you would like to discuss with the GP? Yes/No. If yes, please elaborate.

Pilot testing the prototype

Phase 3: First pilot testing
During the first pilot testing, the questionnaire was continuously revised as the pilot testing was designed and conducted in a stepped process. Based on the patients' experiences with filling out the questionnaire, the response scale was simplified, and instructions were added. The response scale was changed from a five-point Likert scale ('highly agree', 'agree' 'neither agree nor disagree', 'disagree' and 'highly disagree') to a three-point Likert scale ('agree', 'neutral' and 'disagree').

Phase 4: Second pilot testing
During the second pilot testing, the order of the questions was changed into starting with the questions that the patients found easiest to answer. The font type and size was changed, as some patients were visually impaired and found it hard to read the questions in the original layout.

Table 2: Changes made at phase 3-4 during the development of the prototype

Phase	Phase 3<br>1st pilot 1	Phase 4<br>2nd pilot 2
**Revisions**	Simplification of response options. Rewording of difficult questions. Adding an introductory sentence.	Changing the order of the questions. Graphic setup of questionnaire.
**Format after phase**	List of five questions<br>Response scale to a three-point Likert scale (agree, neutral, disagree).	List of five questions<br>Response scale to a three-point Likert scale (agree, neutral, disagree).
**Content after phase**	You will soon visit your GP and talk about your medication. Completing this form will help you prepare for the conversation. At the same time, you will help your GP select the best treatment for you. Please bring the questionnaire at the next appointment with your doctor.<br><br>Do you mostly agree or disagree in the following statements?<br>• I am overall satisfied with my current medication<br>• I sometimes think that I get too much medication.<br>• I experience adverse drug reactions of the medication that bother me significantly.<br>• I think that I might get some medication that I do not need.<br>• Is there something about your medication that you would like to discuss with the GP? Yes/No. If yes, please elaborate.	You will soon visit your GP and talk about your medication. Completing this form will help you prepare for the conversation. At the same time, you will help your GP select the best treatment for you. Please bring the questionnaire at the next appointment with your doctor.<br><br>Do you mostly agree or disagree in the following statements?<br>• I experience adverse drug reactions of the medication that bother me significantly.<br>• I sometimes think that I get too much medication.<br>• I think that I might get some medication that I do not need.<br>• I am overall satisfied with my current medication<br>• Is there something about your medication that you would like to discuss with the GP? Yes/No. If yes, please elaborate.

References

1. Horne R, Weinman J, Hankins M: **The beliefs about medicines questionnaire: The development and evaluation of a new method for assessing the cognitive representation of medication**. *Psychol Health* 1999, **14**(1):1-24.
2. Herborg H, Haugbolle LS, Sorensen L, Rossing C, Dam P: **Developing a generic, individualised adherence programme for chronic medication users**. *Pharm Pract (Granada)* 2008, **6**(3):148-157.

3. Horne R, Hankins M, Jenkins R: **The Satisfaction with Information about Medicines Scale (SIMS): a new measurement tool for audit and research**. *Qual Health Care* 2001, **10**(3):135-140.
4. Jenkins L, Britten N, Stevenson F, Barber N, Bradley C: **Developing and using quantitative instruments for measuring doctor–patient communication about drugs**. *Patient Educ Couns* 2003, **50**(3):273-278.
5. Rovers J, Hagel H: **Self-assessment tool for screening patients at risk for drug therapy problems**. *J Am Pharm Assoc (2003)* 2012, **52**(5):646-652.
6. Snyder ME, Pater KS, Frail CK, Hudmon KS, Doebbeling BN, Smith RB: **Utility of a brief screening tool for medication-related problems**. *Res Social Adm Pharm* 2015, **11**(2):253-264.
7. Atkinson MJ, Sinha A, Hass SL, Colman SS, Kumar RN, Brod M, Rowland CR: **Validation of a general measure of treatment satisfaction, the Treatment Satisfaction Questionnaire for Medication (TSQM), using a national panel study of chronic disease**. *Health Qual Life Outcomes* 2004, **2**:12.
8. Atkinson MJ, Kumar R, Cappelleri JC, Hass SL: **Hierarchical construct validity of the treatment satisfaction questionnaire for medication (TSQM version II) among outpatient pharmacy consumers**. *Value Health* 2005, **8 Suppl 1**:S9-S24.
9. Bharmal M, Payne K, Atkinson MJ, Desrosiers MP, Morisky DE, Gemmen E: **Validation of an abbreviated Treatment Satisfaction Questionnaire for Medication (TSQM-9) among patients on antihypertensive medications**. *Health Qual Life Outcomes* 2009, **7**(1):36.
10. Ruiz MA, Pardo A, Rejas J, Soto J, Villasante F, Aranguren JL: **Development and validation of the "Treatment Satisfaction with Medicines Questionnaire" (SATMED-Q)**. *Value Health* 2008, **11**(5):913-926.
11. Duncan P, Murphy M, Man MS, Chaplin K, Gaunt D, Salisbury C: **Development and validation of the Multimorbidity Treatment Burden Questionnaire (MTBQ)**. *Bmj Open* 2018, **8**(4):e019413.
12. Reeve E, Wiese MD, Hendrix I, Roberts MS, Shakib S: **People's Attitudes, Beliefs, and Experiences Regarding Polypharmacy and Willingness to Deprescribe**. *J Am Geriatr Soc* 2013, **61**(9):1508-1514.
13. Reeve E, Low LF, Shakib S, Hilmer SN: **Development and Validation of the Revised Patients' Attitudes Towards Deprescribing (rPATD) Questionnaire: Versions for Older Adults and Caregivers**. *Drugs Aging* 2016, **33**(12):913-928.
14. Lundby C, Simonsen T, Ryg J, Sondergaard J, Pottegard A, Lauridsen HH: **Translation, cross-cultural adaptation, and validation of Danish version of the revised Patients' Attitudes Towards Deprescribing (rPATD) questionnaire: Version for older people with limited life expectancy**. *Res Social Adm Pharm* 2020.
15. Horne R, Faasse K, Cooper V, Diefenbach MA, Leventhal H, Leventhal E, Petrie KJ: **The perceived sensitivity to medicines (PSM) scale: an evaluation of validity and reliability**. *Br J Health Psychol* 2013, **18**(1):18-30.
16. Katusiime B, Corlett SA, Krska J: **Development and validation of a revised instrument to measure burden of long-term medicines use: the Living with Medicines Questionnaire version 3**. *Patient Relat Outcome Meas* 2018, **9**:155-168.
17. Kang J, Rhew K, Oh JM, Han N, Lee IH, Je NK, Ji E, Lee E, Yoon JH, Rhie SJ: **Satisfaction and expressed needs of pharmaceutical care services and challenges recognized by patients in South Korea**. *Patient Prefer Adherence* 2017, **11**:1381-1388.
18. Cameron KA, Ross EL, Clayman ML, Bergeron AR, Federman AD, Bailey SC, Davis TC, Wolf MS: **Measuring patients' self-efficacy in understanding and using prescription medication**. *Patient Educ Couns* 2010, **80**(3):372-376.
19. Risser J, Jacobson TA, Kripalani S: **Development and psychometric evaluation of the Self-efficacy for Appropriate Medication Use Scale (SEAMS) in low-literacy patients with chronic disease**. *J Nurs Meas* 2007, **15**(3):203-219.

20. De Geest S, Abraham I, Gemoets H, Evers G: **Development of the long-term medication behaviour self-efficacy scale: qualitative study for item development**. *J Adv Nurs* 1994, **19**(2):233-238.
21. Mast R, Ahmad A, Hoogenboom SC, Cambach W, Elders PJ, Nijpels G, Hugtenburg JG: **Amsterdam tool for clinical medication review: development and testing of a comprehensive tool for pharmacists and general practitioners**. *BMC Res Notes* 2015, **8**:642.
22. Pauly A, Wolf C, Mayr A, Lenz B, Kornhuber J, Friedland K: **Effect of a Multi-Dimensional and Inter-Sectoral Intervention on the Adherence of Psychiatric Patients**. *PLoS One* 2015, **10**(10):e0139302.
23. Rogers EA, Yost KJ, Rosedahl JK, Linzer M, Boehm DH, Thakur A, Poplau S, Anderson RT, Eton DT: **Validating the Patient Experience with Treatment and Self-Management (PETS), a patient-reported measure of treatment burden, in people with diabetes**. *Patient Relat Outcome Meas* 2017, **8**:143-156.
24. Schoenmakers TWA, Wensing M, De Smet P, Teichert M: **Patient-reported common symptoms as an assessment of interventions in medication reviews: a randomised, controlled trial**. *Int J Clin Pharm* 2018, **40**(1):126-134.
25. van Summeren JJ, Schuling J, Haaijer-Ruskamp FM, Denig P: **Outcome prioritisation tool for medication review in older patients with multimorbidity: a pilot study in general practice**. *Br J Gen Pract* 2017, **67**(660):e501-e506.
