# Supplementary material for: Involving patients in medicines optimisation in general practice: a development study of the “PREparing Patients for Active Involvement in medication Review” (PREPAIR) tool
Source: BMC Prim Care. 2022 May 20;23:122. doi: 10.1186/s12875-022-01733-8 (PMC9121082; doi:10.1186/s12875-022-01733-8)
Supplement: Supplementary file 4 — Additional file 4: The final PREPAIR tool [file 12875_2022_1733_MOESM4_ESM.pdf]

How do you feel about your medication?

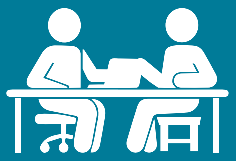

You will soon visit your GP and talk about your medication. Completing this form will help you prepare for the conversation. At the same time, you will help your GP select the best treatment for you.

Please bring the questionnaire at the next appointment with your doctor.

Do you mostly agree or disagree in the following statements?

**I experience adverse drug reactions of the medication that bother me significantly.**	Agree	Neutral	Disagree
**I sometimes think that I get too much medication.**	Agree	Neutral	Disagree
**I think that I might get some medication that I do not need.**	Agree	Neutral	Disagree
**I am overall satisfied with my current medication.**	Agree	Neutral	Disagree

**Is there something about your medication that you would like to discuss with the GP?**	YES	NO

If yes, please elaborate: ______________________________

____

____

AARHUS UNIVERSITY

RESEARCH UNIT FOR GENERAL PRACTICE
Aarhus
